# Comparison of concentrations of chemical species and emission sources PM_2.5_ before pandemic and during pandemic in Krakow, Poland

**DOI:** 10.1038/s41598-022-21012-x

**Published:** 2022-10-01

**Authors:** Anna Rys, Lucyna Samek, Zdzislaw Stegowski, Katarzyna Styszko

**Affiliations:** 1 Faculty of Physics and Applied Computer Science, AGH University of Science and Technology, Al. Mickiewicza 30, 30-059 Kraków, Poland; 2 Faculty of Energy and Fuels, AGH University of Science and Technology, Al. Mickiewicza 30, 30-059 Kraków, Poland

**Keywords:** Environmental sciences, Engineering, Physics

## Abstract

Observations of air pollution in Krakow have shown that air quality has been improved during the last decade. In the presented study two factors affecting the physicochemical characteristic of PM_2.5_ fraction at AGH station in Krakow were observed. One is the ban of using solid fuels for heating purposes and the second is COVID-19 pandemic in Krakow. The PM_2.5_ fraction was collected during the whole year every 3rd day between 2nd March 2020 and 28th February 2021 at AGH station in Krakow. In total 110 PM_2.5_ fraction samples were collected. The chemical composition was determined for these samples. The elemental analysis was performed by energy dispersive X-ray fluorescence (EDXRF) technique, ions analysis was performed by ion chromatography (IC) and black carbon by optical method. In order to identify the emission sources the positive matrix factorization (PMF) was used. The results of such study were compared to similar analysis performed for PM_2.5_ for the period from June 2018 to May 2019 at AGH station in Krakow. The PM_2.5_ concentration dropped by 25% in 2020/2021 in comparison to 2018/2019 at this station. The concentrations of Si, K, Fe, Zn and Pb were lowering by 43–64% in the year 2020/2021 in comparison to 2018/2019. Cu, Mn, Zn and Pb come from mechanical abrasion of brakes and tires while Ti, Fe, Mn and Si are crustal species. They are the indicators of road dust (non-exhaust traffic source). Moreover, the annual average contribution of traffic/industrial/soil/construction work source was reduced in 2020/2021 in comparison to 2018/2019. As well the annual average contribution of fuels combustion was declining by 22% in 2020/2021 in comparison to 2018/2019. This study shows that the ban and lockdown, during COVID-19 pandemic, had significant impact on the characteristic of air pollution in Krakow.

## Introduction

The air pollution is one of the most meaningful environmental problem in cities. The process of fuel combustion and traffic was identified as the greatest contributor to air pollution in urban areas^1^. Epidemiological research shows that both short-term and long-term exposure to air pollution has adverse health effect. It is linked to cardiovascular and respiratory diseases like lung cancer and chronic obstructive pulmonary disease (COPD), that can cause shortening of life expectancy. It is estimated that in 2019 the chronic exposure to fine particulate matter caused 307,000 premature deaths in Europe^2,3^.

The European Union (EU) annual limit values for air pollutants were changed during the last time. So far, the PM_2.5_ annual limit value was 25 μg/m^3^, but from the 1st January 2020 the EU average annual limit value for PM_2.5_ is 20 μg/m^3^^4^. However the World Health Organization (WHO) recommended the average annual limit value as 10 μg/m^3^ and daily limit value as 25 μg/m^3^, but recommendations from 2021 say that the annual limit value should be 5 μg/m^3^ and daily—15 μg/m^3^^1^.

Air pollutants can have natural and anthropogenic origin. To natural sources belong: mineral dust, sea salts, volcanic eruption, forest fires—to anthropogenic sources: industry, combustion from vehicles, combustion from heating in houses during cold season, road dust and biomass burning^5^. To evaluate sources of particulate matter in Krakow receptor model positive matrix factorization (PMF) was used. Such studies were performed for PM_2.5_ fraction for the whole years 2016/2017 and 2018/2019 for the AGH station by Samek et al.^6,7^. In the study^6^ four factors were obtained. To the first factor, two sources were attributed combustion and biomass burning (annual mean contribution was equal to 42.5%), to the second factor, secondary sulphate and nitrate were attributed (annual mean contribution was equal to 30.7%), to the third factor, three sources were attributed, i.e., traffic, soil, and industry (annual mean contribution was equal to 18.7%). Non-identified source contributed 8.2% to PM_2.5_ mass. In the study^7^ also four factors were obtained from PMF analysis. They were attributed to the following sources: soil, traffic/industry, fossil fuel combustion and secondary inorganic aerosols (SIA). Radiocarbon contribution and its sources in Krakow were presented in papers by Zimnoch et al.^8,9^.

During 2020 there were two main aspects which affected the air quality in Krakow. The first is the ban started in September 2019. The second is a COVID-19 pandemic started in March 2020. In March 2020, the COVID-19 lockdown has been introduced. This period lasted to April 2020, but it was announced again for autumn and winter 2020. During this time, the movement of population and traffic decreased significantly. People have never experienced before such limited human activity. Therefore, it is important to assess the impact of the COVID-19 pandemic on air quality.

The aim of our study was physicochemical characterization of PM_2.5_ fraction in Krakow, Poland together with emission sources modelling by receptor model, before and during the ban of solid fuel combustion for residential heating and COVID-19 pandemic event. The samples of PM_2.5_ were collected in 2020/2021 at AGH station in Krakow. Elemental analysis and ion analysis together with black carbon determination were done. Then, Positive Matrix Factorization modelling was used for determination of emission sources. The samples of PM_2.5_ fraction were also collected in the previous 2018/2019 year at the same site and some results were already partially published^7^. However, raw data were used for comparison purposes of the two mentioned years.

## Methodology

### Sampling

**Figure 1:**
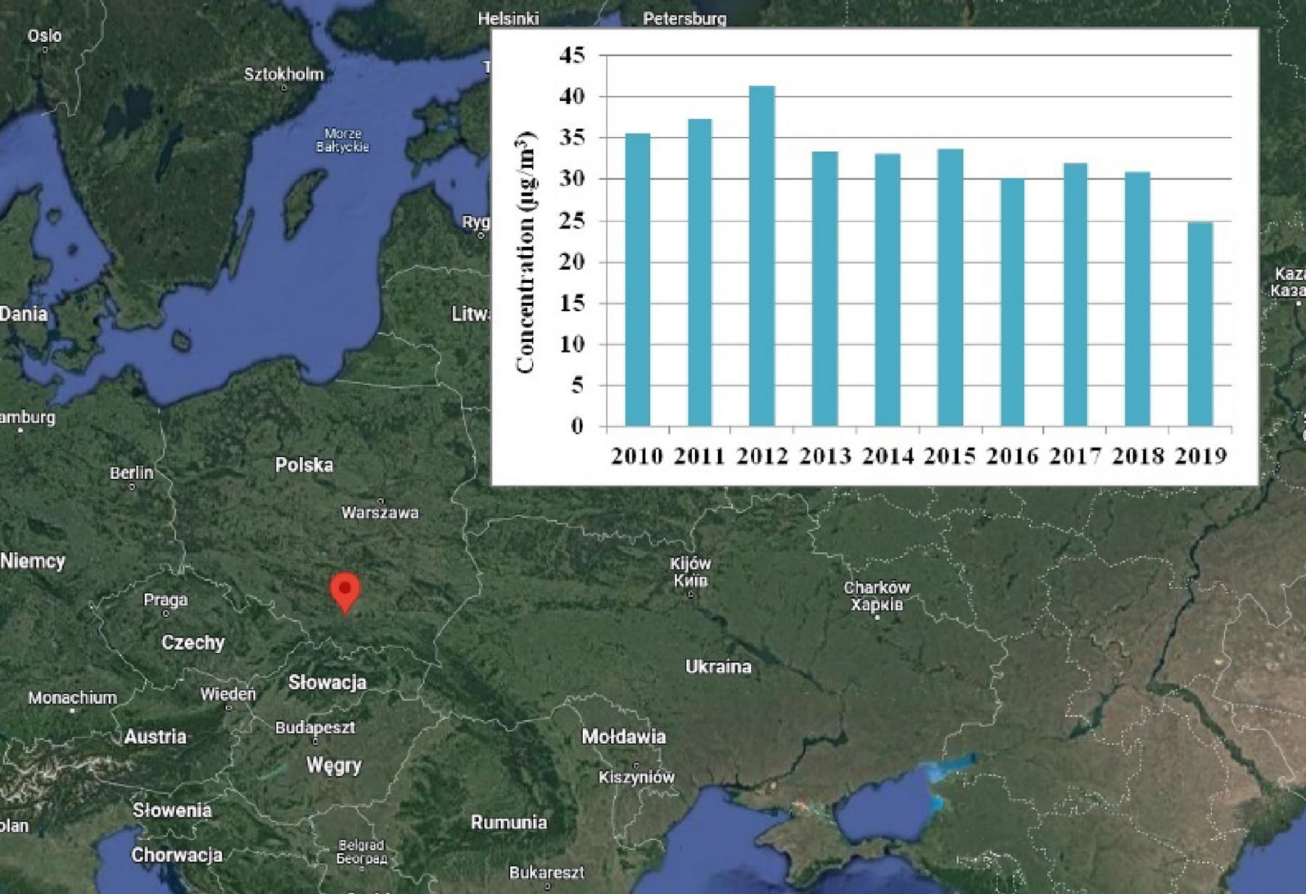
The Map with localization of sampling site in Krakow taken from Google maps and the annual average PM_2.5_ concentration at urban background station in Krakow during the last decade. The graph with concentrations was created based on information available on website https://powietrze.gios.gov.pl/pjp/archives?lang=pl, accessed on 22 February 2022. The GIMP 2.10.24 software was used to combine map and graph with appropriate resolution as Figure.

PM_2.5_ samples were collected at the AGH University of Science and Technology research station in Krakow, Poland. The research station is a typical urban background site with residential and commercial buildings. Next to the station, there are housing estates and a two-lane dual carriageway. The sampling place is about 2 km from the City Centre. Figure 1 presents the map with localization of sampling site and the graph with annual average PM_2.5_ concentrations at the urban background monitoring station in Krakow during the last decade^10^. Sampling was performed over 24 h (i.e., 8:00 a.m. to 8:00 a.m. the next day—110 samples in total) period every 3rd day between 2 March 2020 and 28 February 2021, as annual period. The PM_2.5_ were collected on 46.2 mm diameter PTFE Teflon filters (Whatman) using a low-volume sampler at flow rate of 2.3 m^3^/h. All samples were stored in a refrigerator at − 20 °C before and after analyses.

### Gravimetrical and chemical analyses

The filters were weighed according to PN-EN 12,341 standard before and after exposition (weighed five times). They are to be conditioned before at the temperature 20 ± °C and relative humidity 50 ± 5% for 48 h. Chemical element concentrations (Si, P, S, Cl, K, Ca, Ti, V, Cr, Mn, Fe, Co, Ni, Cu, Zn, As, Br, Rb, Sr and Pb) were analyzed by ED-XRF Spectrometer (energy dispersive X-ray fluorescence). Description of the EDXRF system is in Supplement. Chemical elements such as Ti, V, Cr, Mn, Ni, Cu, As, Rb, Sr had the concentration values above detection limit only for a few samples in the period 2020/2021, and they were excluded from calculation of average values.

Ions concentrations were determined by isocratic ion chromatography on an ICS-1100 instrument (Thermo Scientific) equipped with an autosampler AS-DV. Separations were accomplished using an Ion Pac AS22 (4 × 250 mm) analytical column, (mobile phase: 4.5 mM Na2CO3 + 1.4 mM NaHCO3), and a CS16 (5 × 250 mm) analytical column (mobile phase: 12 mM MSA) for anions and cations, respectively. Samples (25 µL injection volume) were separated with a flow rate of 1.2 mL min–1 of mobile phase. More details about ions analysis methodology was presented in previous paper^7,11^.

Equivalent black carbon (eBC) or light absorbing carbon (LAC) is the most strongly light-absorbing component of particulate matter. It is formed during the incomplete combustion^12,13^. For this study, LAC was identified by Multi-wavelength Absorption Black carbon Instrument (MABI) which was developed by the Australian Nuclear Science and Technology Organization (ANSTO). Description of the MABI is in Supplement.

Positive matrix factorization (PMF) was applied to identification and quantification of the major aerosol sources, using the EPA PMF5.0 software. PMF is a multivariate factor analysis tool which decomposes a matrix of specified sample data into two matrices: factor contributions and factor profiles. These factor profiles require interpretation by the user to identify the source types^14,15^. This method is presented in detail by Paatero and Tapper^16^. More information is in Supplement.

## Results and discussion

### PM_2.5_ concentration

**Figure 2:**
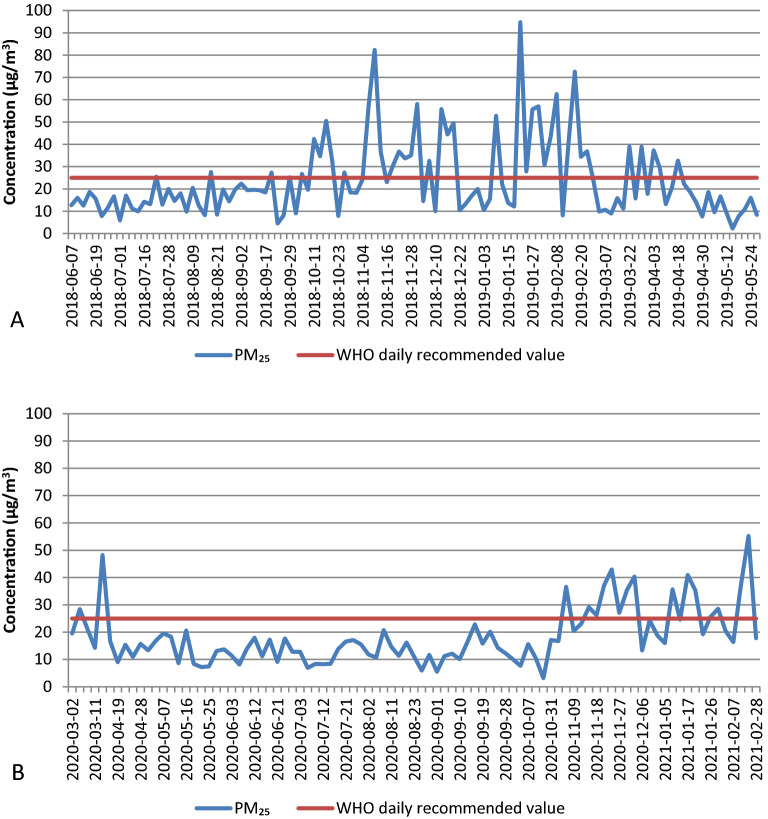
The daily concentration of PM_2.5_ fraction at AGH station in Krakow. (**A**) The values for the year 2018/2019 and (**B**) the values for the year 2020/2021. Red line-WHO recommended daily limit value.

Figure 2 presents the daily variation of PM_2.5_ concentrations in 2018/2019 (A) and 2020/2021 (B). The greatest fluctuations in concentration were observed during cold periods.

**Figure 3:**
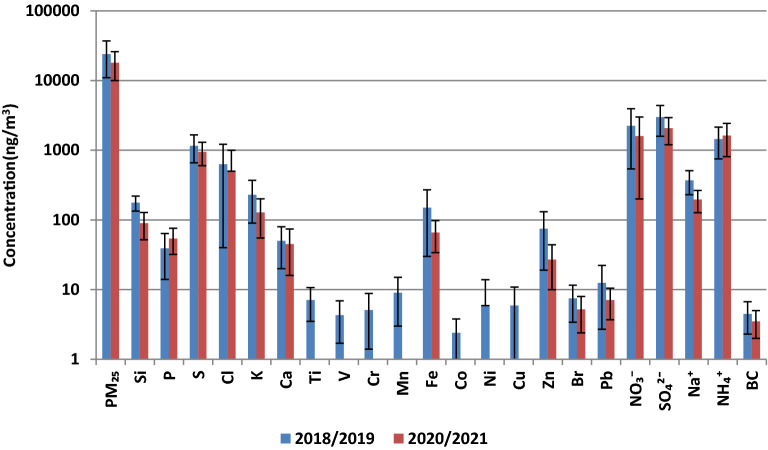
Annual concentration of PM_2.5_, chemical elements, ions and equivalent black carbon with standard deviation.

The annual average PM_2.5_ concentration during the 2020/2021 period was equal to 18 ± 8 μg/m^3^. It means that this annual average value did not exceed the EU annual limit value. The average PM_2.5_ values for the seasons are as follows. The lowest value of PM_2.5_ was in summer 13 ± 4 μg/m^3^ with the range of concentrations: 6–21 μg/m^3^. The highest value of PM_2.5_ concentration was in winter—28 ± 10 μg/m^3^ with the season’s range: 13–55 μg/m^3^. The Fig. 3 and Table 1 present the annual and seasonal PM_2.5_ concentrations for the year 2018/2019 and 2020/2021.

**Table 1:** The seasonal PM_2.5_ (in μg/m^3^), chemical elements (in ng/m^3^), ions (in ng/m^3^) and eblack carbon (in μg/m^3^) concentrations with variability of measurements for the year 2018/2019 and 2020/2021.

Season	Concentration ± SD							
	Spring		Summer		Autumn		Winter	
Year	2019	2020	2018	2020	2018	2020	2019	2020
PM2.5	17 ± 8	17 ± 6	15 ± 5	13 ± 4	28 ± 12	19 ± 9	35 ± 19	28 ± 10
Si	< LLD	93 ± 29	300 ± 92	89 ± 43	< LLD	< LLD	249 ± 94	< LLD
P	< LLD	39 ± 10	103 ± 29	75 ± 27	57 ± 24	56 ± 23	120 ± 28	50 ± 22
S	836 ± 340	810 ± 320	1120 ± 340	960 ± 350	1300 ± 580	870 ± 320	1340 ± 660	1250 ± 410
Cl	358 ± 300	360 ± 340	102 ± 66	32 ± 20	669 ± 540	620 ± 640	1390 ± 710	1010 ± 460
K	139 ± 68	117 ± 54	130 ± 48	65 ± 26	282 ± 120	159 ± 85	320 ± 180	197 ± 75
Ca	30 ± 17	40 ± 17	56 ± 25	62 ± 43	150 ± 64	29 ± 18	47 ± 17	41 ± 31
Ti	7.3 ± 3.6	< LLD	7.0 ± 0.8	< LLD	11 ± 7	< LLD	20 ± 11	< LLD
V	< LLD	< LLD	7.0 ± 2.8	< LLD	< LLD	< LLD	10.1 ± 4.9	< LLD
Cr	< LLD	< LLD	7.5 ± 4.4	< LLD	4.2 ± 1.5	< LLD	10.2 ± 4.3	< LLD
Mn	4.8 ± 2.2	< LLD	7.7 ± 3.2	< LLD	13.1 ± 8.0	< LLD	10.7 ± 4.5	< LLD
Fe	60 ± 37	67 ± 40	154 ± 75	64 ± 26	270 ± 210	68 ± 25	94 ± 52	65 ± 41
Co	0.94 ± 0.46	< LLD	1.70 ± 0.49	< LLD	2.6 ± 1.1	< LLD	3.5 ± 1.8	< LLD
Ni	< LLD	< LLD	4.7 ± 2.5	< LLD	2.18 ± 0.40	< LLD	33 ± 40	< LLD
Cu	1.9 ± 1.0	< LLD	9.8 ± 7.5	< LLD	10.4 ± 6.1	< LLD	7.4 ± 4.2	< LLD
Zn	31 ± 20	27 ± 16	54 ± 32	15 ± 8	136 ± 100	30 ± 16	70 ± 36	42 ± 21
Br	4.8 ± 2.3	5.3 ± 2.3	4.3 ± 1.3	2.54 ± 0.66	7.6 ± 3.6	5.6 ± 3.0	12.6 ± 4.7	8.4 ± 2.6
Rb	0.7 ± 0.2	< LLD	1.11 ± 0.50	< LLD	0.71 ± 0.25	0.86 ± 0.13	2.6 ± 2.8	< LLD
Sr	0.82 ± 0.76	< LLD	1.15 ± 0.46	< LLD	1.18 ± 0.86	< LLD	2.1 ± 2.5	< LLD
Pb	< LLD	7.8 ± 3.3	14.1 ± 4.2	4.3 ± 1.6	22 ± 12	7.4 ± 3.5	22.9 ± 9.3	10.4 ± 3.3
NO_3_^−^	2330 ± 1400	1800 ± 1400	670 ± 280	430 ± 290	2500 ± 1800	1400 ± 980	3300 ± 2000	3400 ± 1400
SO_4_^2−^	2500 ± 1100	1800 ± 750	3050 ± 1200	2000 ± 750	3400 ± 1500	1800 ± 770	3000 ± 1700	2900 ± 1350
Na^+^	360 ± 130	185 ± 55	290 ± 140	157 ± 51	390 ± 170	240 ± 110	410 ± 140	169 ± 39
NH_4_^+^	1220 ± 500	1490 ± 760	960 ± 300	1260 ± 610	1640 ± 810	1580 ± 740	1890 ± 800	2490 ± 750
eBC	3.4 ± 1.1	3.0 ± 1.1	2.7 ± 0.6	2.2 ± 0.5	5.5 ± 2.5	4.2 ± 1.6	6.2 ± 2.7	5.3 ± 1.8

*SD* variability of the concentration during measuring period.

Figure S1 shows the annual and seasonal average PM_2.5_ concentrations for the year 2018/2019 and 2020/2021. Table S1 presents the ratios of PM_2.5_, chemical elements, ions and equivalent black carbon concentrations in 2018/2019 to 2020/2021, respectively. The annual average PM_2.5_ concentration equal to 24 ± 13 μg/m^3^ was observed in 2018/2019 and the ratio PM_2.5_ concentration in 2018/2019 to 2020/2021 was 1.33. During winter 2018/2019 the concentration equal to 35 ± 19 μg/m^3^ was 1.24 times higher than during winter 2020/2021. In summer 2018 the PM_2.5_ concentration equal to 15 ± 5 μg/m^3^ was 1.14 times higher than that value for summer 2020.

### Chemical analyses

Concentrations of chemical elements, ions and eBC were presented in Fig. 3 and Table 1. The sum of the investigated elements was 11% of PM_2.5_ mass and ions 30% of PM_2.5_ mass. During annual sampling period in 2020/2021, the highest annual concentration values were identified for elements such as: S (950 ± 350 ng/m^3^), Cl (500 ± 500 ng/m^3^), Si (90 ± 38 ng/m^3^), K(128 ± 73 ng/m^3^) and ions: SO_4_^2−^ (2070 ± 870 ng/m^3^), NH_4_^+^ (1620 ± 810 ng/m^3^), NO_3_^−^ (1600 ± 1400 ng/m^3^), Na^+^ (196 ± 69 ng/m^3^). The lowest annual concentration values were for Pb (7.1 ± 3.4 ng/m^3^) and Br (5.2 ± 2.8 ng/m^3^).

The annual values of P concentration were slightly higher in 2020/2021 than in 2018/2019, and they were equal to 54 ± 22 ng/m^3^ and 39 ± 25 ng/m^3^ in 2020/2021, and 2018/2019, respectively. However, the values of annual concentrations of such elements like Zn, Fe, Si, K, Pb were higher in 2018/2019 than in 2020/2021. The biggest differences in annual concentration values were for elements for which the ratio “element’s concentration before pandemic” to “element’s concentration during pandemic” ranged from 3 to 2. Such ratios were observed for the following elements: Zn and Fe. Table S1 presents the ratios for elements, ions and eBC for all seasons and annual results for the year 2018/2019 and 2020/2021.

The annual NH_4_^+^ concentration equal to 1620 ± 810 ng/m^3^ was observed during “pandemic and after introducing the ban” and annual NH_4_^+^ concentration before “pandemic and before introducing the ban” was equal to 1450 ± 700 ng/m^3^. The rest of the identified ions (NO_3_^−^, SO_4_^2−^, Na^+^) had higher annual concentration values in 2018/2019 than in 2020/2021.

During summer and autumn higher concentrations of Ca, Zn, Fe and Pb were observed for the year 2018 then 2020. These elements can be connected with construction work, road dust and industry.

Interesting is the fact that a lot of elemental concentration values (S, Ca, Fe, Zn, Br, Cl ) were similar to one another—in spring 2020 to spring 2019.

For the following elements: P, Cl, Br, Pb, Zn, K and Fe higher concentrations were observed in winter 2018/2019 than in winter 2020/2021. The following elements were present in PM_2.5_ in winter 2018/2019: Ti, V, Cr, Mn, Co, Ni, Cu, Rb, Sr and they were not detected in winter 2020/2021. K, Co, Cl, Br and Pb originate mostly from sources which are more active during winter months for example heat generation installations including large and small scale coal/biomass combustion installations^17^.

In winter 2018/2019 and 2020/2021, concentrations of NO_3_^−^ had the highest values from all sampling periods and they were 3300 ± 2000 ng/m^3^ and 3400 ± 1400 ng/m^3^ for winter 2018/2019 and 2020/2021, respectively. Much lower NO_3_^−^ concentrations were observed for summer. They were four and eight times lower than in winter in the year 2018 and 2020, respectively. The Na^+^ concentrations were higher in 2018/2019 than in 2020/2021 for all seasons.

Equivalent black carbon (eBC) concentrations in 2018/2019 were general higher than in 2020/2021. The annual averages show that eBC was 1.29 times higher before pandemic and the ban (4.5 ± 2.2 μg/m^3^) than in pandemic and the ban (3.5 ± 1.5 μg/m^3^). In summer 2018, eBC concentration was 2.7 ± 0.6 μg/m^3^, but in 2020 it was 2.2 ± 0.5 μg/m^3^. In spring the difference was not significant, in 2018 eBC was 3.4 ± 1.1 μg/m^3^ and in 2020 it was 3.0 ± 1.1 μg/m^3^.The highest eBC concentration was in winter 2018/2019 (6.2 ± 2.7 μg/m^3^). The significant difference of eBC concentration occurred in autumn when eBC concentrations were 5.5 ± 2.5 μg/m^3^ in autumn 2019 and 4.2 ± 1.6 μg/m^3^ in autumn 2020.

**Figure 4:**
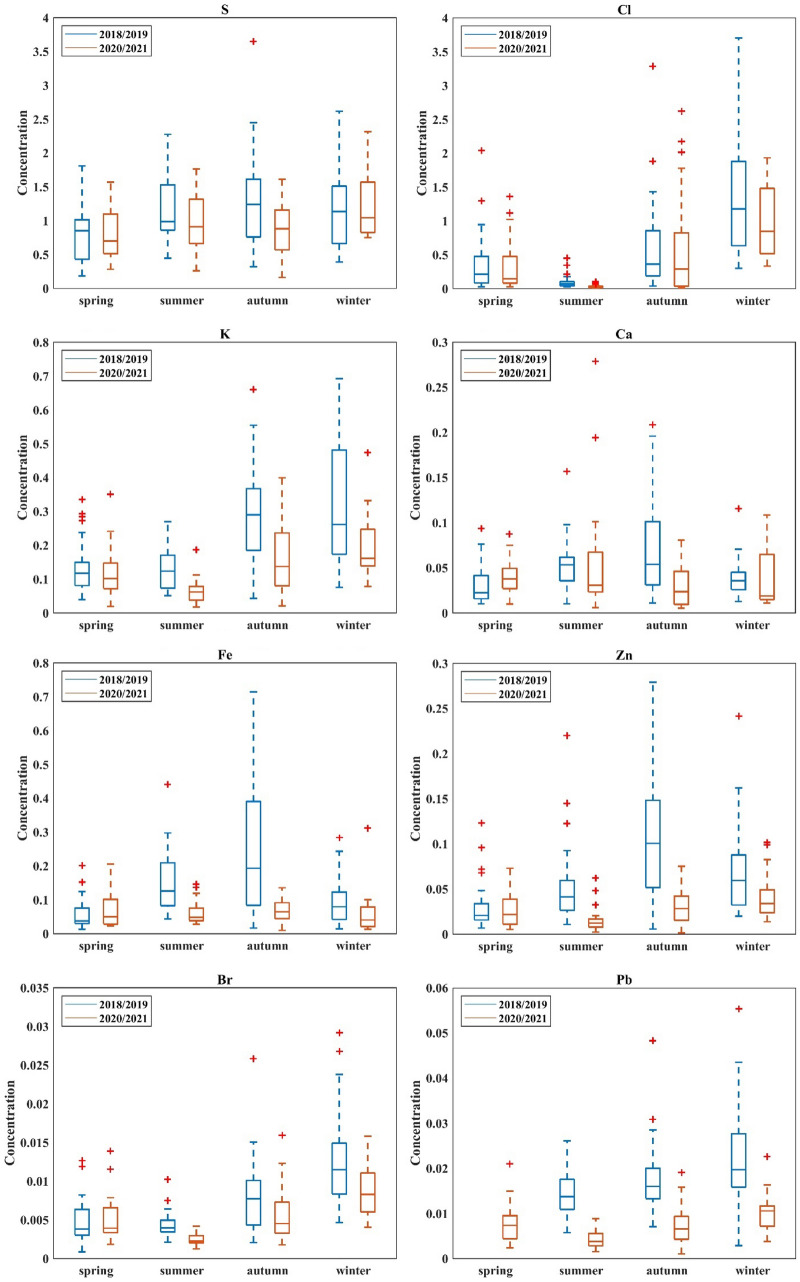
Box plots with interquartile spans and median, minimum, maximum values of elements concentrations for 2018/2019 and 2020/2021.

**Figure 5:**
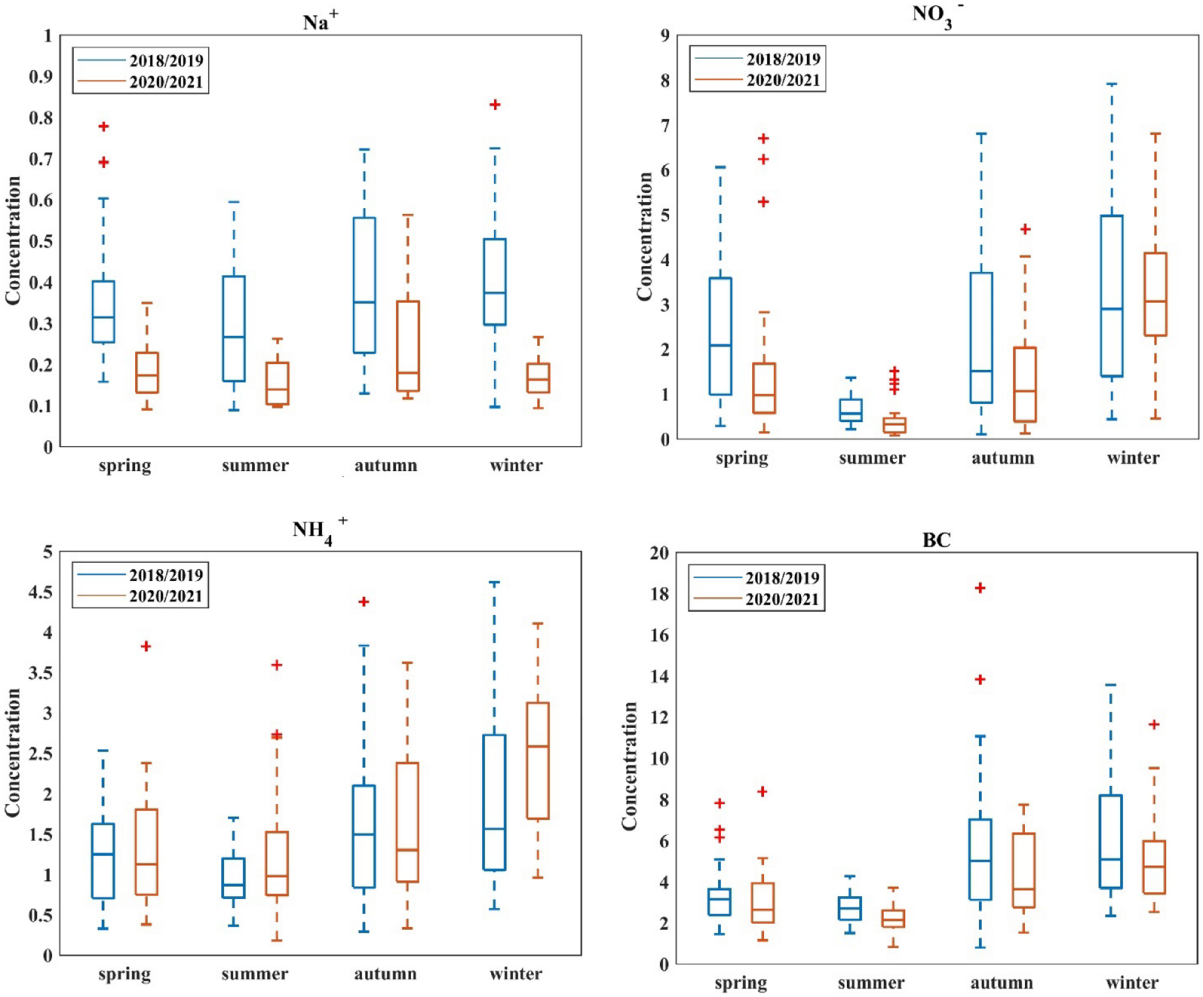
Box plots with interquartile spans and median, minimum, maximum values of ions and eBC concentrations for 2018/2019 and 2020/2021.

The median values with minimum, maximum and interquartile spans were determined for each component analyzed during this study and are presented in Figs. 4 and 5. As can be seen, elements and eBC medians were higher in 2018/2019 than 2020/2021 during summer, autumn and winter. However for spring, lower concentrations of elements were observed in 2018/2019. The median of ion concentrations were lowering in the year 2020/2021 in comparison to 2018/2019.

### Positive matrix factorization

**Figure 6:**
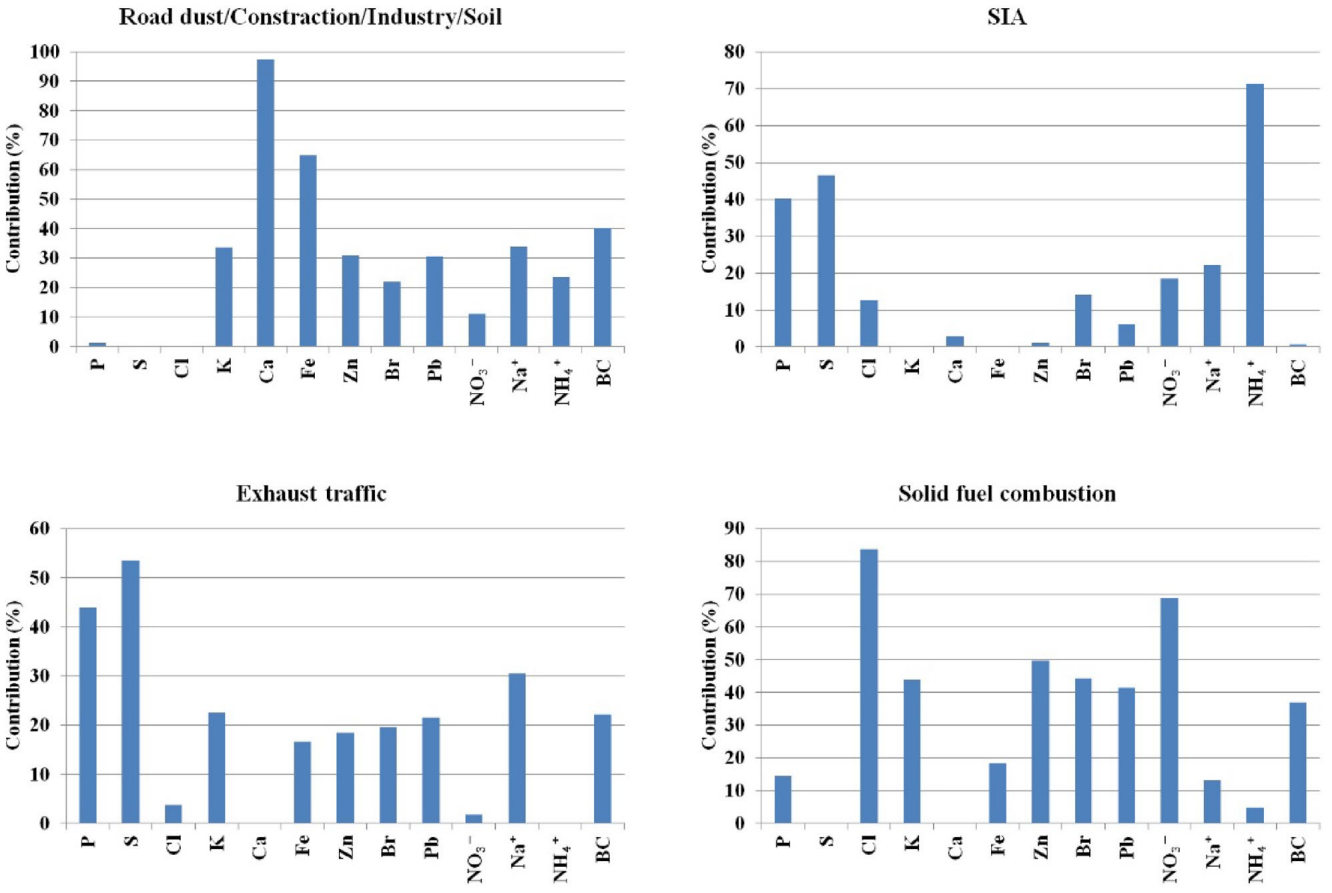
PMF factor profiles for 2020/2021.

**Figure 7:**
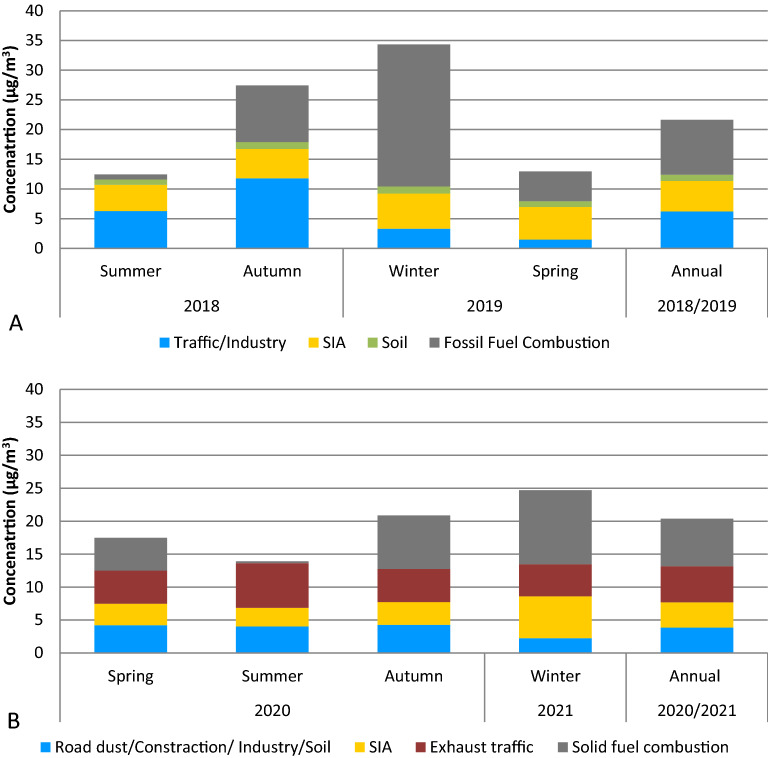
The seasonal and annual contribution of sources to PM_2.5_ mass in μg/m^3^. (**A**) For the year 2018/2019 and (**B**) for the year 2020/2021.

PMF technique was applied to identify sources of pollution and their seasonal and annual contributions to the PM_2.5_ mass for the dataset obtained at the AGH site. The chemical elements, ions and eBC were used for the identification of sources. The factor profiles obtained by the PMF modelling are shown in Fig. 6. The contributions of particular sources to PM_2.5_ mass are presented in Fig. 7 and Tables S2 and S3. Figure S2 shows relation between modelled by PMF and measured PM2.5 concentration (y = 0.94x and R = 0.98).

Table S2 shows the contributions of sources to PM_2.5_ mass in percent. Four factors were obtained from PMF modeling and the following sources were attributed to them: road dust/construction work/industry/soil, exhaust traffic and solid fuel combustion as well as secondary inorganic aerosols.

#### Factor 1—road dust/construction work/industry/soil

This factor was dominated by Ca (97% of mass), Fe (65% of mass), K (34% of mass), Zn (31% of mass), Pb (31% of mass), Br (22% of mass) and Na^+^ (34% of mass), NH_4_^+^ (24% of mass), BC (40% of mass). Ca indicates construction work and crustal element, while Fe and Pb can be connected to industry^13–16^. Zn is a main additive to lubricant oil. Moreover, Fe and Zn are associated with tires and brake ware, tailpipe emission, corrosion of vehicular. Zn and Pb can be also emitted from asphalt pavement^18,19^. The contribution of Na^+^ to this factor was also observed at the same urban site in the previous study. In 2018/2019, the concentration for Na^+^ was 0.37 μg/m^3^ and was higher than in this study where Na^+^ is equal to 0.20 μg/m^3^^5,20^. EBC is mainly emitted by the incomplete combustion of carbon containing fuels^17^.

The contributions of this factor to PM_2.5_ mass in spring, summer and autumn 2020/2021 were very similar to each other and they were equal to 26% (4.2 µg/m^3^), 32% (4.0 µg/m^3^) and 22% (4.3 µg/m^3^), respectively. The contribution of this factor to PM_2.5_ mass was the lowest during winter and it was equal to 8% (2.2 µg/m^3^). The same phenomena was observed in 2018/2019 when the contribution of Traffic/Industrial source to PM_2.5_ mass was the lowest also during winter and it was equal to 7% (3.3 µg/m^3^). Factor 1 from the year 2020/2021 can be roughly compared to two factors Traffic/Industry and Soil from the year 2018/2019. The annual outcomes show that the contributions of Traffic/Industry and Soil to PM_2.5_ mass were 24% and 4% in 2018/2019 (before pandemic and introducing the ban), respectively and they were higher than the contribution of Factor 1 to PM_2.5_ mass (21%) in 2020/2021 (during pandemic and after introducing the ban). However, the annual contributions of these factors to PM_2.5_ in units were 7.2 μg/m^3^ and 3.9 μg/m^3^ in 2018/2019 and 2020/2021, respectively. The seasonal contributions of two factors (Traffic/Industry and Soil) to PM_2.5_ mass in μg/m^3^ were equal to 2.5 µg/m^3^, 7.2 µg/m^3^ and 12.9 µg/m^3^ in spring, summer and autumn 2018/2019, respectively. The presented results show that the contribution of Traffic/Industry/Construction work/Soil was declining during pandemic 2020/2021 in comparison to the year 2018/2019. The biggest decreasing of the contribution of these sources to PM_2.5_ mass was observed in autumn followed by summer and winter.

#### Factor 2—secondary inorganic aerosols (SIA)

This factor is represented by NH_4_^+^ (71% of mass), S (47% of mass), P (40% of mass) and Na^+^ (22% of mass), NO_3_^−^ (18% of mass).

The factor mainly characterized by NH_4_^+^ and S and NO_3_^−^ was attributed to secondary inorganic aerosols^18,21,22^.

The observed contributions of this factor to PM_2.5_ mass were 20% (3.3 µg/m^3^), 22% (2.8 µg/m^3^), 18% (3.5 µg/m^3^) and 23% (6.4 µg/m^3^) in spring, summer, autumn and winter 2020/2021, respectively. Study performed for the year 2018/2019 shows that the SIA contributions to PM_2.5_ mass were 32% (5.5 µg/m^3^), 29% (4.4 µg/m^3^), 18% (5.0 µg/m^3^) and 13% (5.9 µg/m^3^) in spring, summer, autumn and winter, respectively. The lockdown in the year 2020 caused by COVID-19 pandemic together with introducing the ban of using solid fuels for heating purposes affected the decline of contributions of SIA source to PM_2.5_ mass in each season of the year. The annual SIA contributions were 20% (5.2 µg/m^3^), and 21% (3.8 µg/m^3^) in 2018/2019 and 2020/2021, respectively.

#### Factor 3—exhaust traffic

Exhaust traffic was identified by S (53% of mass), P (44% of mass), K (23% of mass), Pb (22% of mass), Br (20% of mass), Zn (18% of mass), Fe (17% of mass), Na^+^ (31% of mass) and eBC (22% of mass).

Zn is a main additive to lubricant oil. eBC is produced by the incomplete combustion of carbon containing fuels—as for diesel vehicles^23,24^. The presence of eBC in this factor highlights the primary origin of fuel combustion in vehicle engines. Small amounts of sulphur components exist in gasoline, different forms of sulphur such as sulphates, sulphides or oxysulphides can also be formed in three-way catalytic converters^17^. Br is also indicating motor vehicles^25^. Near-road aerosols may comprise combustion-derived carbonaceous nuclei or ultrafine particles with trace amounts of vaporized S and P and metal constituents such as Ca, K, Fe, and Al from the fuel, the lubricating oils, or their additive^26,27^. The Ca, K, and Fe observed in the fine and ultrafine near-highway particles possibly vaporized from the lubricating oil^26,27^.

This factor represents 30% (5.5 µg/m^3^) of the total fine particle mass during annual analysis in 2020/2021. Seasonal trends indicate that summer was the period with the highest average contribution with 54% (6.8 µg/m^3^), however during other seasons the contributions were similar: spring—30% (5.0 µg/m^3^), autumn—26% (5.0 µg/m^3^), and the lowest in winter—18% (4.9 µg/m^3^). We did recognize such source in the year 2018/2019.

#### Factor 4—solid fuel combustion

This factor was identified by Cl (84% of mass), Zn (50% of mass), Br (44% of mass), K (44% of mass), Pb (41% of mass), NO_3_^−^ (69% of mass), and eBC (37% of mass). Zn, Cl and Pb are tracers of residential coal combustion^28–30^. The high impact of K is reported to originate from biomass burning^31^. NO_3_^−^ and eBC are produced by incomplete combustion of solid fuels^30,32,33^. The contribution of primary species like eBC in this source means that in Krakow some part of atmospheric PM is emitted directly from different solid fuels. The source profile shows also high contributions of secondary inorganic aerosols. It is noticeable strong seasonal variability of this factor during the year. This factor is attributed as residential combustion of both primary and secondary origin. The contributions of solid fuel combustion to PM_2.5_ mass were 41% (11.2 µg/m^3^), 42% (8.1 µg/m^3^), 30% (5.0 µg/m^3^) and 2% (0.3 µg/m^3^) in winter, autumn, spring and summer 2020/2021. The contributions of fossil fuel combustion to PM_2.5_ mass were 53% (23.9 µg/m^3^), 35% (9.5 µg/m^3^), 29% (5.0 µg/m^3^), and 6% (0.8 µg/m^3^) in winter, autumn, spring and summer 2018/2019, respectively. Strong reducing of the contribution of this source was observed in winter and it can be connected with introducing the ban of using coal and wood for heating purpose in September 2019 in Krakow. The comparison of annual result for the year 2020/2021 to our previous study from 2018/2019 shows that the contribution of this factor before the ban and pandemic was 36% (9.2 µg/m^3^) and it was higher than during introducing the ban and pandemic (40%–7.2 µg/m^3^).

The main innovation of this study is the proof that introducing ban of using solid fuel combustion for heating purpose works and such decision is lowering PM_2.5_ concentrations and contribution of combustion source to PM_2.5_ mass radically. Such results can convince decision makers and sceptics to implement similar regulations in different sites all over the world.

### Statistical analysis

The Wilcoxon rank sum test was used to test whether there were significant differences in medians for PM_2.5_, elements and ions as well as sources concentrations during two periods of time: 2018/2019 and 2020/2021.

The Wilcoxon rank sum test is a non-parametric test that requires no specific distribution on the measurements (like a normal distribution for instance).

This analysis tests the null hypothesis that data in x and y are samples from continuous distributions with equal medians, against the alternative that they are not, where x and y are samples from 2018/2019 and 2020/2021. The result h = 1 indicates a rejection of the null hypothesis, and h = 0 indicates a failure to reject the null hypothesis at the 5% significance level.

The h-values of the test results were shown in Tables 2 and 3. Tables 2 and 3 present results for statistic test. Two observations are very interesting, however. For the most of components, it was observed the h = 0 in spring. For Fe, Zn, Br, Pb and Na^+^ (summer, autumn, winter) the hypothesis with equal medians can be rejected. Moreover, similar results are for Factor 1-Road dust/Construction/Industry/Soil (in summer and autumn). The PMF analysis has shown that these elements are indicators of this factor, so the statistic confirmed earlier discussion.

**Table 2:**
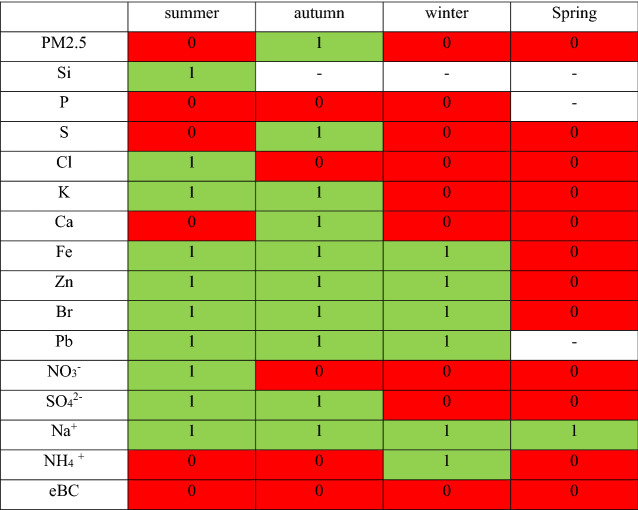
Wilcoxon rank sum test results for PM_2.5_, elements and ions: 1 indicates a rejection of the statement that medians are equal; 0 indicates a failure to reject the statement that medians are equal.

**Table 3:**
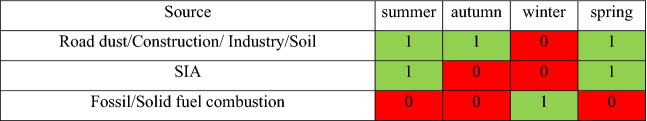
Wilcoxon rank sum test results for sources: 1 indicates a rejection of the statement that medians are equal; 0 indicates a failure to reject the statement that medians are equal.

## Conclusion

The study was performed for the year 2020/2021 when lockdown caused by COVID-19 pandemic together with the ban of using solid fuel for heating purposes in Krakow were introduced. The obtained results were compared to similar study performed for the same location for the year 2018/2019, the period before pandemic and introducing the ban in Krakow, Poland. The annual PM_2.5_ concentration dropped in 2020/2021 in comparison to 2018/2019 by about 25%. The annual concentrations of the most of chemical species were lower in 2020/2021 then in 2018/2019. The traffic related elements (Fe, Zn, Cu, Br, Pb) concentrations were lower in 2020/2021 then 2018/2019. Statistical analysis shows difference between Fe, Zn, Br and Pb concentration in 2020/2021 and 2018/2019. The contribution of road dust/construction work/industry/soil was declining during pandemic and after introducing the ban in Krakow in 2020/2021 in comparison to 2018/2019. During lockdown 2020/2021 the car traffic and the movement of people were reduced. The restrictions lasted from 23 March 2020 to 20 April 2020 and in autumn 2020 (from 17th October) and lasted with different intensity. Strong lowering of solid fuel combustion contribution (by about 53%) was observed for winter 2020/2021 in comparison to winter 2018/2019. Such event can be connected to introduction of the ban of solid fuel combustion for heating purposes in Krakow in September 2019. The results of our study can convince decision makers and sceptics to implement similar decision all over the world.

## Supplementary Information

Supplementary Information.

## Data Availability

The datasets used and/or analysed during the current study available from the corresponding author on reasonable request.
