# Supplementary material for: Comparison of concentrations of chemical species and emission sources PM2.5 before pandemic and during pandemic in Krakow, Poland
Source: Sci Rep. 2022 Oct 1;12:16481. doi: 10.1038/s41598-022-21012-x (PMC9526202; doi:10.1038/s41598-022-21012-x)
Supplement: Supplementary file 1 — Supplementary Information. [file 41598_2022_21012_MOESM1_ESM.docx]

**Supplement**

Comparison of Concentrations of Chemical Species and Emission Sources PM_2.5_ Before Pandemic and During Pandemic in Krakow, Poland.

Anna Rys^(1)^, Lucyna Samek^(1)^, Zdzislaw Stegowski^(1)^ and Katarzyna Styszko^(2)^

^(1)^ Faculty of Physics and Applied Computer Science, AGH University of Science and Technology, Al. Mickiewicza 30, 30-059 Krakow, Poland

^(2)^ Faculty of Energy and Fuels, AGH University of Science and Technology, Al. Mickiewicza 30, 30-059 Krakow, Poland

Corresponding author: Lucyna.Samek@fis.agh.edu.pl

Methodology

Chemical analysis

The spectrometer consists of 3 kW Mo-anode water cooled X-ray tube with line focus (300 μm Be exit window, take off angle 6°, effective source size 0.4 mm x 0.8 mm), silicon drift detector (SDD active area 70 mm^2^ collimated to 50mm^2^, FWHM = 124 eV at 5.9 keV, 12.5 μm Be window), 8-positions sample changer integrated with secondary target holder with switchable secondary targets. The measurement geometry is defined by three axes perpendicular to each other: (1) X-ray source-secondary target, (2) secondary target-sample, (3) sample-detector. The acquisition process was controlled with the use of in-house developed LabView program. The X-ray tube was operated at 55 kV/30 mA exciting Ni and Mo secondary targets. Chemical elements such as: Si, P, S, Cl were detected with Ni secondary target; the measurement time for a single sample was 1 000 seconds. Chemical elements (such as K, Ca, Ti, V, Cr, Mn, Fe, Co, Ni, Cu, Zn, As, Br, Rb, Sr and Pb) were detected with Mo secondary target and the measurement time for one sample was 2 400 seconds. The specimens are placed in the plate with 8 positions with the automatic changer of samples. The measurements were performed under atmosphere air pressure.

Black carbon analysis

The MABI measures light absorption at seven different wavelengths: 405 nm(UV), 465 nm, 525 nm, 639 nm, and infrared 870 nm, 940 nm, and 1050 nm. The eBC mass concentration (μg/m^3^) was calculated based on the Equation 1:

|  | $eBC \left( ngm^{-3} \right)=\frac{{10}^{5}\cdot A}{\varepsilon\cdot V}\cdot\ln\left[ \frac{I_{0}}{I} \right]$ | (1) |
| --- | --- | --- |

Where parameter $A$is the collection area of the exposed filter ${(cm}^{2})$ and $V$ is the volume of sampled air on the filter $(m^{3})$, $I_{0}$ is the measured light transmitted through the blank filter, I is the measured light transmitted through the sample, $\varepsilon$ is the mass absorption coefficient ($m^{2}g^{-1}$). The value of mass absorption coefficient was used as $\varepsilon=6.036 m^{2} g^{-1}$ at 639 nm.

PMF analysis

In this study, the following species: chemical elements, ions and eBC were used for the identification of PM_2.5_ emission sources. Species were categorized as “bad”, “weak” and “strong”. The classification was performed on the base of the signal-to-noise ratios calculated from the concentrations and uncertainties. For example, the species which have the value of uncertainty smaller than the measured signal, was classified as “strong” and “weak” is when the uncertainty is large in relation to the signal. “Weak” data are taken with less weight in PMF modeling [1]. More details about this calculation and method were presented in previous papers [2, 3]. For the year 2020/2021, S, Cl, K, Ca, Fe, Zn, Br, Pb, NO_3_^-^, NH_4_^+^, BC were categorized as “strong”, and P, Na^+^ were categorized as “weak” and PM_2.5_ as a total variable was categorized as “weak”. Other element and ion concentrations, in many samples, were less than the detection limits and they were not taken in PMF modeling. The Q(robust) was 6783 and Q(True) was 10 769.

**Results**


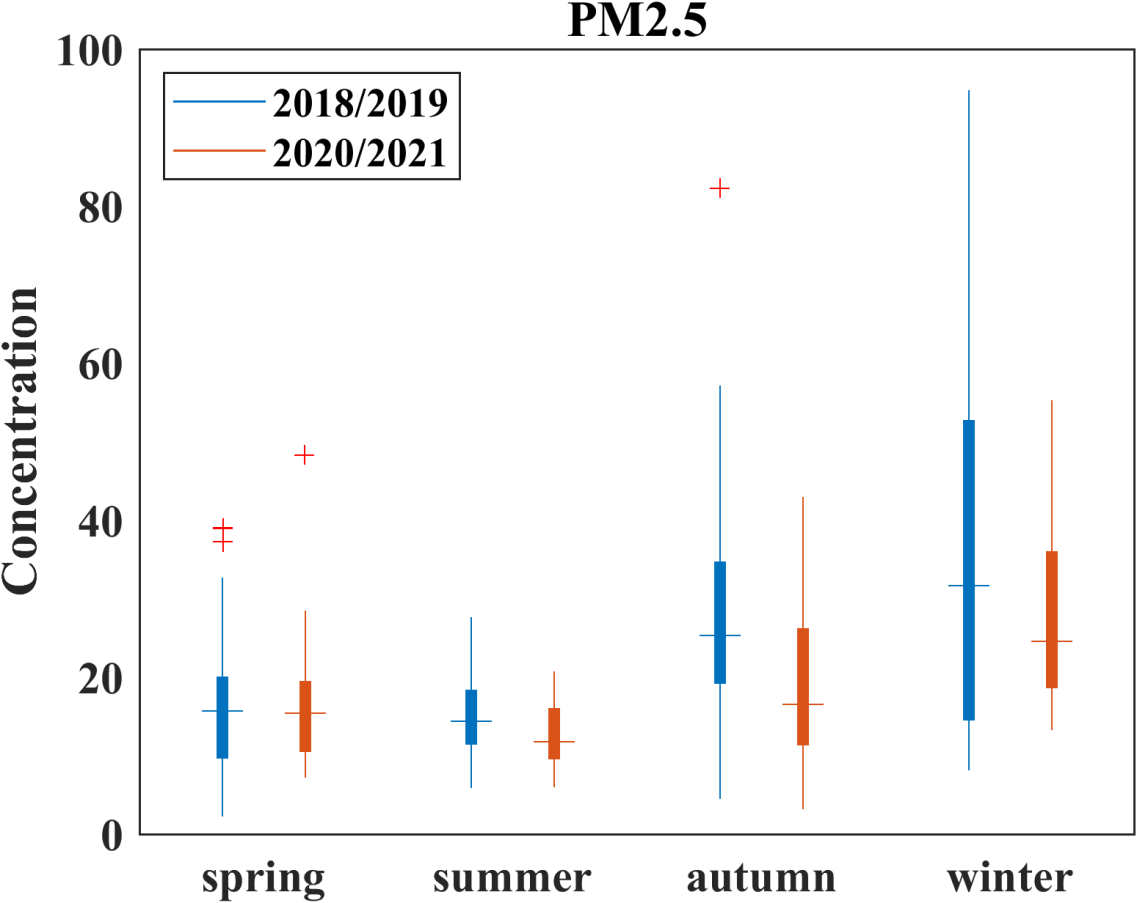


Figure S 1. Seasonal concentration of PM_2.5_ in μg/m^3^.

Table S 1.The Ratio of PM_2.5_ and chemical species concentrations before pandemic (2018/2019) to during pandemic (2020/2021).

| Element/ion | summer | autumn | winter | spring | annual |
| --- | --- | --- | --- | --- | --- |
| PM2.5 | 1.14 | 1.47 | 1.24 | 1.00 | 1.33 |
| Si | 3.38 | - | - | - | 1.97 |
| P | 1.37 | 1.02 | 2.39 | - | 0.72 |
| S | 1.17 | 1.50 | 1.07 | 1.03 | 1.22 |
| Cl | 3.18 | 1.07 | 1.38 | 0.99 | 1.31 |
| K | 2.00 | 1.77 | 1.62 | 1.19 | 1.80 |
| Ca | 0.90 | 5.17 | 1.15 | 0.75 | 1.11 |
| Fe | 2.41 | 3.97 | 1.44 | 0.90 | 2.27 |
| Zn | 3.60 | 4.53 | 1.67 | 1.15 | 2.78 |
| Br | 1.70 | 1.36 | 1.49 | 0.90 | 1.46 |
| Pb | 3.28 | 2.96 | 2.20 | 0.00 | 1.76 |
| NO_3_^-^ | 1.55 | 1.73 | 0.98 | 1.37 | 1.42 |
| SO_4_^2-^ | 1.54 | 1.85 | 1.03 | 1.37 | 1.44 |
| Na^+^ | 1.85 | 1.62 | 2.43 | 1.95 | 1.89 |
| NH_4_ ^+^ | 0.76 | 1.04 | 0.76 | 0.82 | 0.89 |
| eBC | 1.23 | 1.31 | 1.17 | 1.13 | 1.29 |

Table S 2. Seasonal and annual contributions of sources to PM_2.5_ in %.

| Source | Spring | Summer | Autumn | Winter | Annual |
| --- | --- | --- | --- | --- | --- |
| 2018/2019 | | | | | |
| Traffic/Industry | 9 | 41 | 43 | 7 | 24 |
| SIA | 32 | 29 | 18 | 13 | 20 |
| Soil | 6 | 6 | 4 | 3 | 4 |
| Fossil Fuel Combustion | 29 | 6 | 35 | 53 | 36 |
| 2020/2021 | | | | | |
| Road dust/Construction/ Industry/Soil | 26 | 32 | 22 | 8 | 21 |
| SIA | 20 | 22 | 18 | 23 | 21 |
| Exhaust traffic | 30 | 54 | 26 | 18 | 30 |
| Solid fuel combustion | 30 | 2 | 42 | 41 | 40 |

Table S 3. Seasonal and annual contributions of sources to PM_2.5_ in μg/m^3^

| Source | Spring | Summer | Autumn | Winter | Annual |
| --- | --- | --- | --- | --- | --- |
| 2018/2019 | | | | | |
| Traffic/Industry | 1.5 ± 1.5 | 6.3 ± 3.6 | 11.8 ± 9.0 | 3.3 ± 2.6 | 6.2 ± 5.6 |
| SIA | 5.5 ± 2.2 | 4.4 ± 1.7 | 5.0 ± 3.5 | 5.9 ± 4.1 | 5.2 ± 2.9 |
| Soil | 1.0 ± 0.4 | 0.9 ± 0.5 | 1.1 ± 0.6 | 1.2 ± 0.4 | 1.0 ± 0.5 |
| Fossil Fuel Combustion | 5.0 ± 3.6 | 0.8 ± 1.0 | 9.5 ± 7.9 | 23.9 ± 9.4 | 9.2 ± 8.9 |
| 2020/2021 | | | | | |
| Road dust/Construction/ Industry/Soil | 4.2±2.0 | 4.0±1.1 | 4.3±1.8 | 2.2±1.3 | 3.9±1.7 |
| SIA | 3.3±1.9 | 2.8±1.9 | 3.5±2.2 | 6.4±2.1 | 3.8±2.4 |
| Exhaust traffic | 5.0±1.2 | 6.8±2.5 | 5.0±1.9 | 4.9±2.1 | 5.5±2.1 |
| Solid fuel combustion | 5.0±4.4 | 0.3±0.2 | 8.1±7.0 | 11.2±4.7 | 7.2±5.9 |

**Figure S 2.** Modelled by PMF and measured PM_2.5_ concentration.

Reference

1. Karagulian, F.; Belis, C.A. Enhancing source apportionment with receptor models to foster the air quality directive implementation. Int. J. Environ. Pollut. 2012, 50, 190–199.

2. Samek L, Turek-Fijak A, Skiba A, Furman P, Styszko K, Furman L, Stegowski Z. 2020 Complex Characterization of Fine Fraction and Source Contribution to PM2.5 Mass at an Urban Area in Central Europe. *Atmosphere (Basel).* **11**. (doi:10.3390/atmos11101085).

3. United States Environmental Protection Agency. 2014 EPA Positive Matrix Factorization (PMF) 5.0 Fundamentals and User Guide.
